# The complete chloroplast genome of *Secale sylvestre* (Poaceae: Triticeae)

**DOI:** 10.1007/s13353-021-00656-x

**Published:** 2021-08-15

**Authors:** Lidia Skuza, Romain Gastineau, Anna Sielska

**Affiliations:** 1 Institute of Biology, University of Szczecin, PL-71-415, Szczecin, Poland; 2 Centre for Molecular Biology and Biotechnology, University of Szczecin, PL-71-415, Szczecin, Poland; 3 Institute of Marine and Environmental Sciences, University of Szczecin, PL-70-383 Szczecin, Poland

**Keywords:** Chloroplast genome, Rye, *Secale sylvestre*, Sequencing

## Abstract

*Secale sylvestre* is a wild species of rye, morphologically distinct from domestic species. To draw comparisons between species based on molecular features, it is important to have high-quality sequences, especially in the case of organellar genomes. For such reason, the complete chloroplast genome of *Secale sylvestre* Host introd. no. 6047 will provide useful data for ecological, agricultural, and phylogenetic purposes. Here we present the complete, annotated chloroplast genome sequence of *Secale sylvestre* Host introd. no. 6047. The genome is 137116 base pair (bp) long. It is the first complete chloroplast genome that can be used as a reference genome for further analysis. The genome can be accessed on GenBank with the accession number (MW557517).

## Introduction

*Secale* is a small but very diverse genus from the tribe *Triticeae* (family *Poaceae*). It includes annual, perennial, self-pollinating, and open-pollinating, cultivated, weedy, and wild species of various morphologies. The genus *Secale* includes for now four species whose phylogenetic relationships have not been fully determined (GRIN, http://www.arsgrin.gov). This causes a significantly reduction of progress in rye breeding that can be enriched with functional traits derived from wild rye species. In the genus, the wild species *Secale sylvestre* Host (1809) is singularized by several genetic peculiarities (Bennett and Smith 1976; Singh and Röbbelen 1977; Shang et al. 2006; Zhou et al. 2010; Skuza et al. 2019a).

Among the 8 chloroplast genome of *Secale* spp. available on GenBank, none is complete strictly speaking, with the second copy of the inverted repeat (IR) missing all the time, and with the exception of *Secale cereale* KC912691, they all display several ambiguous and non-attributed bases, rending it difficult to perform accurate SNPs comparisons. Thus, we presume that analysis of the complete chloroplast genome sequences of *Secale* spp., starting with *S. sylvestre*, will be useful and cost-effective for evolutionary and phylogenetic studies, as was suggested by our previous studies (Doyle and Doyle 1990; Skuza et al. 2019b).

## Material and methods

Seeds of *Secale sylvestre* Host introd. no. 6047 were obtained from the Botanical Garden of the Polish Academy of Sciences in Warsaw. Total DNA was extracted from young sprouts following Doyle and Doyle (Bankevich et al. 2012). Sequencing took place in BGI Shenzhen’s facilities on a. An amount of ca. 40 million clean 100 bp paired-end reads was obtained and assembled using SPAdes 3.14.0 (Gordon and Green 2013) with a k-mer of 85. The contigs corresponding to the chloroplast genome were joined together using Consed (Tillich et al. 2017). Annotations were performed with the help of GeSeq (Katoh and Standley 2013) and manually curated.

## Results and discussion

The genome is 137116 bp long (Table 1). The large single copy (LSC) is 81132 bp long, the short single copy (SSC) is 12820 bp long, and the IR is 21582 bp long. No ambiguous bases were found in the genome.

**Table 1:** Overview of the data files/data sets

Label	File types (file extension)	Data repository and identifier (DOI or accession number)
*Secale sylvestre* chloroplast, complete genome	FASTA, annotated GBK	MW557517

As stated above, SNP calling type of analysis was rendered difficult by the presence among 7 out of 8 of the other available genomes of numerous non-attributed bases. Instead, analyses focused on the presence of indels, which were found to be common and often phylogenetically informative (e.g., Chen et al. 2016). To do so, chloroplast genomes were partitioned by sub-units, aligned using MAFFT 7 (Kumar et al. 2018), and then visualized using MEGAX (Mo et al. 2020).

Results provided evidences of the strong proximity between *S. sylvestre* Host introd. no. 6047 and *Secale strictum* voucher R 1108 (KY636137). A total of 16 indels were found to be common between these two strains that discriminate them from all others (KC912691, KY636135, KY636136, KY636132, KY636134, KY636133, KY636138). This number is not very high compared to the results presented by other authors (e.g., Shaw et al. 2007), but considering that only two species were analyzed, the result was very satisfactory. The size of these indels ranges from 2 to 36 bp. Among these indels, 13 of were found in intergenic sequences (*rpl32* – *tRNA-L*; *psaC* – *ndhE*; *rrn16* – *trnI-GAU*; *atpH* – *atpF*; *psaA* – *ycf3*; *trnT-UGU* – *trnL-UAA*; *trnF-GAA* – *ndhJ*; *atpB* – *rbcL*; *ycf4* – *cemA*; *trnP-UGG* – *psaJ*; *psaJ* – *rpl33*; *clpP* – *psbB*; *rpl16* – *rps3*). It is worth being underlined that the last three indels occurred in intronic sequences, one inside a tRNA (intron *trnK-UUU*), two inside protein-coding genes (intron *rps16*; intron *petD*), a feature that received recent attention (Liu et al. 2019; Chen et al. 2020), especially for the purpose of genetic distinction between closely related species.

### Limitations

The protocol itself showed no limitation, as it allowed to obtain complete and non-ambiguous genome sequence. However, far more clean genome sequences are needed in order to describe the most reliable molecular markers for species identification and phylogeny, especially for what concerns SNPs.

## Data Availability

The genome has been deposited on GenBank with the accession number MW557517. It is also available on Zenodo with the following link: http://doi.org/10.5281/zenodo.4537281.
